# Editorial: Exploring pollen microbiome: implications for plant physiology, crop improvement and human allergies

**DOI:** 10.3389/fmicb.2025.1717890

**Published:** 2025-11-10

**Authors:** Yalavarthi Nagaraju, Sodimalla Triveni, Talati Srinvas Rao, Massimilano Cardinale

**Affiliations:** 1 Scientist-B, CSB-Central Sericultural Research and Training Institute, Berhampore, WB, India; 2 Department of Microbiology and Bio-energy, Professor Jayashankar Telangana State Agricultural University, Hyderabad, India; 3 Genetics and Plant Breeding, Acharya N.G. Ranga Agricultural University, Lam, Guntur, India; 4 Department of Biological and Environmental Sciences and Technologies - DiSTeBA, University of Salento, Lecce, Italy

**Keywords:** *Pantoea* sp., vertical transmission, pollen microbiome, crop improvement, human allergy

The transition of plants from their vegetative to reproductive phase is marked by flowering, a complex, tightly regulated process critical for pollination, seed production, and species survival (Barrett, 2008).

Plant-associated microorganisms—including bacteria, fungi, and viruses—are transmitted across plant generations and ecosystems through both vertical and horizontal pathways, influencing plant fitness, growth, stress tolerance, and disease susceptibility. Both beneficial and pathogenic microbes exploit these transmission modes, often interacting with insects, pollen, soil, and air as dispersal vectors.

Pollen grains are nutrient-rich microhabitats containing amino acids, lipids, and coenzymes that support microbial growth. Beneficial endophytes are transferred to ovules via pollen and support nitrogen fixation, seedling vigor, and early growth (Madmony et al., 2005). Pollen-associated microbial communities are influenced by pollinators, wind, and anthropogenic activities, thus creating a dynamic interface of microbial transfer.

Volatile organic compounds (VOC) profiles, pollinator diversity, nectar composition, and temporal variations play a central role in the assemblage of microorganisms on pollen. Furthermore, trichomes and cuticular structures on petals and anthers provide microhabitats that retain both humidity and nutrients, creating niches for microbial persistence. These structural traits, therefore, act as ecological filters that shape the microbiome at both structural and functional levels.

The successful establishment begins with the attachment to pollen grains, which provide an initial substrate and favorable habitat for microbial colonization. Pollen morphological features significantly influence the diversity and persistence of pollen-associated microbes. The degree of surface ornamentation, ranging from smooth to highly reticulate or spiny exines, shapes microbial interactions by offering microhabitats where bacteria and fungi can attach and evade desiccation (Herrera et al., 2013). The survival of genera such as *Acinetobacter, Pseudomonas, Sphingomonas*, and *Methylobacterium* is facilitated by their metabolic versatility, while others like *Bacillus* and *Staphylococcus* persist through endospore formation or biofilm production (Ambika Manirajan et al., 2016).

Considering both the critical role of pollen for plant biology and the recognized importance of the plant-associated microbiome for plant fitness, we decided to propose this research topic on the pollen microbiome. The idea was to stimulate research on the topic as well as allow for the emergence of new concepts on the structure and function of the pollen microbiome, its role on plant ecology and crop productivity, and its contribution to human allergies.

This research topic received seven manuscripts, four of which were accepted for publication. However, despite the limited number of manuscripts (which may be due to the highly specific topic), the research topic has at time of writing received ~16,000 views and ~3,700 downloads. This demonstrates a notable interest in this new and relevant topic within the broader context of plant microbiome research.

Two research articles and two opinion articles were published, indicating that both scopes were fulfilled (new research/studies and new ideas/concepts). One research article (Khalaf et al.) compared different high-throughput technologies to characterize the maize pollen microbiome (which has never been analyzed before) and identified the most probable main inhabitant: the *Pantoea* genus (specifically the species *ananatis*). This is a very interesting discovery, in light of the fact that *Pantoea* is among the taxa that were previously identified as stable cereal seed colonizers (Rahman et al., 2018) and were recently demonstrated to be transmitted from seeds to the seeds of the next plant generation by inoculation of Green Fluorescent Protein (GFP)-tagged strains in wheat (Sanz-Puente et al., 2025). This highlights a possible link between the pollen and seed microbiome, as discussed in the opinion paper from Cardinale and Schnell.

The pollen microbiome acts as a double-edged sword, providing essential ecological functions while posing significant risks to plant, pollinator, and human health. Beneficial microbial taxa facilitate plant–pollinator interactions, enhance pollen viability, and contribute to ecosystem resilience. However, under specific environmental stresses or agricultural practices, these microbial communities can shift toward pathogenicity, leading to adverse outcomes. Several plant pathogens exploit pollen as a transmission vector, compromising plant reproductive efficiency and yield. For example, *Erwinia amylovora*, the causal agent of fire blight in both *Malus domestica* (apple) and *Pyrus communis* (pear), has been documented to use insect-mediated pollen dispersal to infect flowers (Rivest et al., 2024). Interestingly, Shrestha et al. demonstrated that the pollen-associated microbiome acts as an effective biocontrol agent against the *Fusarium* pathogen, the causative agent of Gibberella ear rot (GER). This research highlights how we still lack a real comprehension of the complex interactions governing and driving the ecology of pollen microbiomes. More research will be needed to enable us to correctly place pollen-associated microbes within the one-health/one-biosecurity frameworks to sustainably link agricultural applications, environmental safety, and animal/human health (Scherman et al.).
